# Coverage of indoor residual spraying for malaria control and factors associated with its acceptability in Nasarawa State, North-Central Nigeria

**DOI:** 10.11604/pamj.2019.33.84.13212

**Published:** 2019-06-06

**Authors:** Hannatu Janada Dimas, Nasir Mohammed Sambo, Muhammed Sani Ibrahim, Ike Oluwapo Oyeneye Ajayi, Patrick Mboya Nguku, Olufemi Olamide Ajumobi

**Affiliations:** 1 Nigeria Field Epidemiology and Laboratory Training Programme, Abuja, Nigeria; 2 Nigeria Security and Civil Defense Corps, Abuja, Nigeria; 3 Department of Community Medicine, Ahmadu Bello University, Zaria, Nigeria; 4 Department of Epidemiology and Medical Statistics, University of Ibadan, Ibadan, Nigeria; 5 African Field Epidemiology Network, Abuja, Nigeria; 6 National Malaria Elimination Programme, Abuja, Nigeria

**Keywords:** Coverage, indoor residual spraying, malaria, prevention and control, utilization, acceptability, Nigeria

## Abstract

**Introduction:**

Indoor residual spraying (IRS) is among the major vector control strategies recommended for endemic populations by the World Health Organization (WHO). The success of IRS requires high coverage which is dependent on its acceptability. In Nigeria, IRS pilots have been ongoing and rejection has been a major setback to its coverage. We assessed coverage of IRS and determined factors associated with its acceptability in Nasarawa Eggon district, Nasarawa state, Nigeria

**Methods:**

A cross-sectional survey involving 409 households selected using multi-stage sampling was carried out. Trained data collectors administered pre-tested structured questionnaire to collect data on socio-demographic characteristics of household heads or their representatives, their perceptions on IRS and factors associated with IRS acceptability. Descriptive, bivariate and multivariate analyses were done at 5% level of significance.

**Results:**

Majority of respondents were male (79.7%) and married (82.6%), and their mean age was 36.4 ± 13.3 years. Coverage of IRS was 99.3%. However, only 82.6% of those who previously accepted IRS were willing to accept it in again. Factors independently associated with acceptability were perceived effectiveness of IRS (aOR = 21.8; 95%CI = 6.9-68.8) and lower household cost of malaria prevention after IRS (aOR = 5.0; 95%CI = 1.1-21.8)

**Conclusion:**

IRS coverage in the communities studied met WHO minimum standard of 85%. However, for similar results to be achieved in future, acceptability must be promoted by providing information on its effectiveness and its ability to reduce household cost of malaria prevention.

## Introduction

In 2006 alone, there were 214 million malaria cases and 438,000 malaria-related deaths with the highest burden being in sub-Saharan Africa [1]. Effective control of malaria requires a multi-pronged approach; vector control through indoor residual spraying (IRS) inclusive. Indoor residual spraying refers to application of insecticides to inner surfaces of dwellings to repel and/or kill vector mosquitoes that come in contact/perch on such surfaces. The main insecticides used for IRS are dichlorodiphenyltrichloroethane (DDT) and pyrethroids (deltamethrin and cypermethrin). Indoor residual spraying in large scale was introduced in 1946, later strengthened in the early 1960s [2, 3]. This intervention was scaled down in the late 1960s due to neglect of the eradication commitment perceived globally [2]. After cessation of IRS, transmission and mortality rates increased in the intervention areas. IRS was first implemented in Nigeria, in Garki district of the Federal Capital Territory with an associated significant reduction in malaria transmission and an improvement in infant and child mortality [4]. There has been a renewed interest in implementing IRS programmes as a key component of malaria control since 2005. The WHO produced a position statement on the application of IRS for malaria control in 2006 [5]. This highlighted IRS as being one of the primary vector control interventions for reducing and interrupting malaria transmission and recommended that it should be a major component of national malaria control strategies in areas where it is feasible and can be implemented effectively. This singular statement by WHO has resulted in many countries opting to include IRS in their national malaria control strategies.

The success of malaria control interventions requires high coverage and utilization at individual and community levels [6]. However, coverage of IRS is dependent on the perceived benefits of the intervention with regard to its effectiveness against the vector and extent of undesired side effects [7] which also influence the acceptability of the intervention. Community acceptability of interventions is requisite for effective implementation of vector control programmes. This is principally relevant with IRS which requires that at least 85% of households in target communities should be sprayed. It is therefore essential to understand the community beliefs which will enable better implementation of the vector control. Most studies on people's perceptions, acceptability and practices about malaria in Africa have focused on acceptability of drugs and insecticide treated nets (ITN) while published information on reception of IRS are usually limited to reports on coverage levels or acceptability among communities that had not experienced IRS. We conducted a community survey to determine the coverage of IRS, acceptability of IRS, and factors associated with acceptability of IRS.

## Methods

**Study setting:** Nassarawa Eggon Local Government Area (LGA) of Nasarawa state, north-central Nigeria is a semi-urban area with farming as main occupation. Nassarawa Eggon is generally hilly and rocky, with numerous rivers and streams that empty into the Benue River. It is in the Sudan savannah belt with high rainfall and intense malaria transmission. Thus, malaria cases are seen all year round with peaks recorded at the beginning and towards the end of the rainy season which spans from March to October. The LGA which has rural and semi-urban settings are divided into three development areas. As at the time of the study, four rounds of IRS have been conducted in two of the 13 LGAs with support from United States President's Malaria Initiative (PMI), namely Nassarawa Eggon and Doma. Other malaria control activities ongoing in the state include free LLIN distribution, malaria testing and treatment and intermittent preventive treatment (IPT) for pregnant women.

**Study population and design:** a household based cross-sectional community survey was conducted, sampling household heads, 18 years and above, residing in selected households in identified settlements prior to the last IRS spray round, irrespective of IRS status of households. Persons who were too sick or too old to comprehend and respond to questions from interviewers were excluded.

**Sample size and sampling method:** sample size was calculated using the formula for single proportion based on prevalence of 41% [8] with 5% precision level and 10% adjustment for non-response. A minimum of 409 households was calculated required for the study. A total of 420 households were selected for the study shared equally among all 14 settlements that represented the 14 wards of the LGA. We used multi-stage sampling technique. In the first stage, Nasarawa Eggon LGA was selected, using balloting, from the two LGAs (Nasarawa Eggon and Doma) that had IRS in Nasarawa state. In the second stage, simple random sampling by balloting was used to select one settlement from each of the 14 wards in Nasarawa Eggon. At the settlement level, bottle spinning was done at the site considered to be the centre of the settlement to determine the starting point. The first house was chosen by balloting, and then contiguous houses were visited until the sample size for each settlement was reached. In the selected house, all households were identified. Where there was more than one eligible household in a house, one of them was selected for interview using balloting. On completion of the interview in one house, the interviewer exited that house and moved to the next house. This process continued until the required sample size of 30 for each settlement was reached. Any household that refused to participate or did not have a representative was skipped.

**Data collection:** the study was part of a larger research submitted as a dissertation to the Ahmadu Bello University, Zaria, Nigeria. A structured interviewer administered-questionnaire was used for the data collection. The questionnaire had sections which sought information on the socio-demographic characteristics of the respondents, knowledge on malaria transmission and preventive measures, history of febrile illnesses within the household before and after spraying periods, perceived benefits of IRS. The perception and beliefs on malaria and IRS were also explored. Six data collectors were recruited from residents of the study communities who understood the local language. They were trained on how to administer the questionnaire and the details of roles and responsibilities of each data collector. They also practiced interviews using role play. Questionnaires were pre-tested among 30 households in three settlements not among the study sample. The pretesting of questionnaires also served as an opportunity for field practice to the research assistants because it was done under supervision. The completed questionnaires were examined on the field on daily basis by the team lead for completeness and consistency.

**Data analysis:** data was entered, cleaned and analyzed using Microsoft excel and Epi-Info version 3.5.4. Data was summarized to obtain frequencies, means and proportions. Association between categorical variables were tested using odds ratios and 95% confidence intervals. Multiple logistic regressions were performed to determine factors independently associated with acceptability of IRS at p< 0.05.

**Ethical clearance:** ethical clearance was obtained from the Research Ethics Committee of Nasarawa State Ministry of Health. Informed consent was obtained from the respondents and confidentiality was maintained. Codes were used to identify respondents and completed questionnaires were kept securely. Data was stored in password-enabled computer.

## Results

A total of 409 respondents were successfully interviewed out of the 420 selected, giving a response rate of 97.4%. Majority of respondents were male 325 (79.7%), married 338 (82.6%), and aged 20-39 years 202 (49.4%) with mean age of 36.4±13.3 years (Table 1). Almost all, 406 (99.3%) of the respondents had IRS in their households out of which 323 (82.6%) were willing to accept IRS in the future. Respondents that accepted IRS did so for several reasons; to kill mosquitoes (55.7%) or to protect family from malaria (37.7%). None was forced to accept the intervention. Side effects were not noticed by 271 (68.8%) of respondents. Among those who noticed side effects, 93 (75.6%) observed non-health-related effects such as bad smell of insecticide, insecticide staining walls and creation of disorder due to spraying processes. About half 207 (52.3%) of the respondents who had IRS reported waiting for 2 to 5 hours after spraying before entering household while 8 (2%) had to wait for > 5 hours. More than half (69.1%) of respondents perceived IRS to be effective. On bivariate analysis, acceptability of IRS showed statistically significant association with age, main occupation, presumption about effectiveness of spraying and number of household fever episodes after IRS (Table 2, Table 3). After controlling for confounders, those who perceived IRS to be effective were about 22 times more likely to accept IRS and those who had lower cost of malaria prevention after IRS were about 5 times more likely to accept IRS. However, age, main occupation and occurrence of fever after IRS were not independently associated with acceptability of IRS (Table 4).

**Table 1:** Socio-demographic characteristics of respondents

Demographic characteristics	Frequency	Percent (%)
**Age**		
<19	3	0.7%
20-39	202	49.4%
40-59	164	40.1%
>60	40	9.8%
**Sex**		
Male	325	79.7%
Female	83	20.3%
**Marital status**		
Divorced	5	1.2%
Married	338	82.6%
Unmarried	44	10.8%
Widow/widower	22	5.4%
**Main Occupation**		
Employed	119	29.1
Farmer	166	40.6
House wife	21	5.1
Others*	36	8.8
Small scale business	33	8.1
**Status in household**		
Head of HH	307	75.1
Spouse of HHH	64	15.6
Others**	38	9.3
**Highest level of education**		
Non	74	18.1
Others***	10	2.5
Primary	73	17.9
Secondary	118	28.9
Tertiary	133	32.6

* These were business owners, salary earners, unemployed

** These were other representatives of household head

*** These were vocational schools, skill acquisition training

**Table 2:** Acceptability of indoor residual spraying by socio-demographic characteristics of respondents

Demographic characteristics	Acceptability of IRS		OR (95%CI)
	Yes n (%)	No n (%)	
Age			
**≥39**	169 (87.6)	24 (12.4)	2.0 (1.2-3.5)
**<39**	154 (77.8)	44 (22.2)	
Sex			
**Male**	265 (83.3)	53 (16.7)	1.1 (0.6-2.0)
**Female**	67 (81.7)	15(18.3)	
Marital status			
**Married**	270 (83.3)	54 (16.7)	1.3 (0.7-2.6)
Unmarried**	53 (79.1)	14 (20.9)	
Highest educational level			
**Secondary and above**	197 (81.7)	44 (18.3)	1.2 (0.7-2.0)
**Primary and below**	126 (84.0)	24 (16.0)	
Main occupation			
**Farmer**	123 (76.9)	200 13.4)	2 (1.3-3.3)
Others*	31 (86.6)	37 (23.1)	
Status in household			
**Head of household**	244 (83.3)	49 (16.7)	1.2 (0.7-2.2)
**Others**^+^	79 (80.6)	19 (19.4)	

* These were business owners, salary earners, unemployed.

+ These were spouses of household head, other representatives of household head.

** These were single, widowed, separated, divorced

IRS = indoor residual spraying

**Table 3:** Acceptability of indoor residual spraying by malaria-related and spraying-related factors

Factors	Acceptability of IRS		Odds ratio(95%CI)
	Yes n(%)	No n(%)	
***Malaria related factors***			
Household fever episodes after IRS			
**Less**	238(93.7)	16(6.3)	9.6(5.2-17.8)
**Not less**	79(60.8)	51(39.2)	
Cost of malaria prevention after IRS			
**Lower**	261(91.9)	23(8.1)	8.8(3.0-25.9)
**Not lower**	9(56.3)	7(43.8)	
Use of bed nets in household			
**No**	89(85.6)	15(14.4)	1.3(0.7-2.2)
**Yes**	234(81.5)	53(18.5)	
***Spraying related factors***			
IRS presumed to be effective in….			
**Yes**	260(97.0)	8(3.0)	32(14.5-70.3)
**No**	61(50.4)	60(49.6)	
Waiting duration after spraying			
**≥2 hours**	173(83.6)	34(16.4)	1.2(0.7-2.0)
**<2 hours**	145(81.5)	33(18.5)	
Reason for refusal			
**Non-health**	310(82.9)	64(17.1)	3.2(0.9-11.8)
**Health**	6(60)	4(40)	
Health related side effects noticed			
**No**	256(83.7)	50(16.3)	1.4(0.7-2.5)
**Yes**	67(78.8)	18(21.2)	

CI =confidence interval**;** IRS = indoor residual spraying

**Table 4:** Factors associated with acceptability of indoor residual spraying among respondents

Factors	Adjusted Odds Ratio	95% Confidence Interval	p-value
**Age**			
>39	2.3	0.9-7.6	0.07
≤39	1		
**Main occupation**			
Farming	2.0	0.7-5.8	0.18
Others	1		
**IRS Presumed to be effective**			
Yes	21.8	6.9-68.8	<0.0001
No	1		
**Fever episodes after IRS**			
Not less	2.5	0.9-7.1	0.09
Less	1		
**Cost of malaria prevention after IRS**			
Lower	5.0	1.1-21.8	0.03
Not lower	1		

IRS = indoor residual spraying

## Discussion

This study observed IRS coverage that is in agreement with the World Health Organization recommendation of more than 80% coverage in targeted communities [5]. The high level of coverage of IRS in this study is similar to the 98.7% documented in the end of spray report of the PMI African Indoor Residual Spraying (AIRS) Project [9]. It is also similar to the 95.3% observed by West *et al.* in Tanzania in a survey conducted following a round of IRS [10]. Because none of the households reported being forced to accept IRS, the high coverage of IRS observed in our study could imply high acceptability among the households prior to the spraying, following the initial advocacy, communication and social mobilization embarked upon by PMI/AIRS. However, studies have reported less than a third coverage in Ethiopia and about 41% in Mozambique [6, 8]. The high acceptability of IRS in this study is similar to the finding of studies in Mexico and Tanzania where majority of the households welcomed future spraying [11, 12]. However, it is noteworthy that the acceptability of future IRS was lower than its coverage. This is possibly because, as observed in this study, some households who had allowed spraying were no longer willing to accept the intervention. Because of such lower acceptability compared to coverage, despite awareness of its effectiveness in malaria prevention and control, future efforts should target the development of insecticides with less side effects [13]. As a short-term measure, the usual pre-spraying advocacy, communication and social mobilization done among the community members should be sustained for longer periods after the spraying. More than half of respondents perceived IRS to be effective with only about a third reporting that it was not effective similar to findings from Kenya [14]. This perception among the residents could have been because they experienced reduction in mosquitoes and/or cases of fever or malaria in the households following IRS. Previous studies in different communities have already reported reduction in prevalence of malaria following the introduction of IRS [1, 15, 16]. This study also found that those who perceived IRS to be effective were nine times more likely to accept it. This is in keeping with what was obtained in another study in Uganda [7]. This is not unexpected because, generally, an intervention is more likely to be accepted by persons involved where they perceive it to be of benefit to their health [17]. Respondents who spent less on malaria prevention after the IRS were more likely to accept IRS. Again, the perceived benefit of the intervention in this case was responsible for its acceptability [17]. Previous studies have shown that introducing an IRS programme causes reduction in malaria-related suffering including expenditures incurred [18-20]. Moreover, economic studies have shown that resources are always limited, and any activity that would lead to lower cost and lower spending would almost always be expressly welcomed [21, 22]. Contrary to findings of a study in Uganda where respondents with secondary education and above were more likely to accept IRS, this study did not find association between level of education and acceptability of IRS [10].

## Conclusion

Coverage of IRS within households in study area was high, though a slightly lower percentage of households were willing to accept IRS in the future. Majority of the respondents perceived IRS to be effective. The major factors associated with acceptability of IRS include perception of IRS to be effective and experience of lower household cost of malaria prevention following IRS. Efforts should be made to improve sensitization especially after IRS round in order to ensure that all households who received IRS do not only remain willing to accept it in the future, but even serve as peer educators and mobilisers of fellow community members. Such efforts should focus especially on sharing information about the effectiveness of IRS and its ability to reduce the cost of malaria prevention among households.

### What is known about this topic

- IRS is effective for malaria control;
- High coverage is necessary for IRS to be effective;
- IRS coverage is dependent on acceptability among households.

### What this study adds

- Coverage of IRS in the study area meets the WHO target of 85%;
- A household that has accepted IRS may not automatically accept it again in the future;
- Willingness to accept IRS in future is dependent on its perceived personal and economic benefits.

## Competing interests

The authors declare no competing interests.

## References

[1] WHO Malaria: World Malaria Report 2013.

[2] CDC Indoor Residual Spraying.

[3] Africa Indoor Residual Spraying Projet Indoor Residual Spraying.

[4] Padonou GG, Gbedjissi G, Yadouleton A, Azondekon R, Razack O, Oussou O (2012). Decreased proportions of indoor feeding and endophily in Anopheles gambiae s.l. populations following the indoor residual spraying and insecticide-treated net interventions in Benin (West Africa). Parasit Vectors.

[5] WHO (2006). Malaria: Indoor residual spraying. Use of indoor residual spraying for scaling up global malaria control and elimination (archived).

[6] Gobena T, Berhane Y, Worku A (2013). Women's knowledge and perceptions of malaria and use of malaria vector control interventions in Kersa, eastern Ethiopia. Glob Health Action.

[7] Ediau M, Babirye JN, Tumwesigye NM, Matovu JK, Machingaidze S, Okui O (2013). Community knowledge and perceptions about indoor residual spraying for malaria prevention in Soroti district, Uganda: a cross-sectional study. Malar J.

[8] Munguambe K, Pool R, Montgomery C, Bavo C, Nhacolo A, Fiosse L (2011). What drives community adherence to indoor residual spraying (IRS) against malaria in Manhiça district, rural Mozambique: a qualitative study. Malar J.

[9] Hlongwana KW, Mabaso ML, Kunene S, Govender D, Maharaj R (2009). Community knowledge, attitudes and practices (KAP) on malaria in Swaziland: A country earmarked for malaria elimination. Malar J.

[10] West PA, Protopopoff N, Rowland M, Cumming E, Rand A, Drakeley C (2013). Malaria Risk Factors in North West Tanzania: The Effect of Spraying, Nets and Wealth. PLOS ONE.

[11] Rodríguez AD, Penilla RP, Rodríguez MH, Hemingway J, Trejo A, Hernández-Avila JE (2006). Acceptability and perceived side effects of insecticide indoor residual spraying under different resistance management strategies. Salud Pública México.

[12] Gimnig JE, Otieno P, Were V, Marwanga D, Abong'o D, Wiegand R (2016). The Effect of Indoor Residual Spraying on the Prevalence of Malaria Parasite Infection, Clinical Malaria and Anemia in an Area of Perennial Transmission and Moderate Coverage of Insecticide Treated Nets in Western Kenya. PLoS ONE.

[13] Toxipedia Toxipedia is resting.

[14] Kleinschmidt I, Schwabe C, Shiva M, Segura JL, Sima V, Mabunda SJ, Coleman M (2009). Combining Indoor Residual Spraying and Insecticide-Treated Net Interventions. Am J Trop Med Hyg.

[15] Pluess B, Tanser FC, Lengeler C, Sharp BL (2010). Indoor residual spraying for preventing malaria. Cochrane Database Syst Rev.

[16] Okumu FO, Moore SJ (2011). Combining indoor residual spraying and insecticide-treated nets for malaria control in Africa: a review of possible outcomes and an outline of suggestions for the future. Malar J.

[17] Janz NK, Becker MH (1984). The Health Belief Model: a decade later. Health Educ Q.

[18] Conteh L, Sharp BL, Streat E, Barreto A, Konar S (2004). The cost and cost-effectiveness of malaria vector control by residual insecticide house-spraying in southern Mozambique: a rural and urban analysis. Trop Med Int Health.

[19] Goodman CA, Mnzava AE, Dlamini SS, Sharp BL, Mthembu DJ, Gumede JK (2001). Comparison of the cost and cost-effectiveness of insecticide-treated bednets and residual house-spraying in KwaZulu-Natal, South Africa. Trop Med Int Health TM IH.

[20] Guyatt HL, Kinnear J, Burini M, Snow RW (2002). A comparative cost analysis of insecticide-treated nets and indoor residual spraying in highland Kenya. Health Policy Plan.

[21] Kernick DP (2003). Introduction to health economics for the medical practitioner. Postgrad Med J.

[22] Jimoh A, Sofola O, Petu A, Okorosobo T (2007). Quantifying the economic burden of malaria in Nigeria using the willingness to pay approach. Cost Eff Resour Alloc.

